# Status and influencing factors of artificial intelligence readiness among nursing students

**DOI:** 10.3389/fmed.2026.2007016

**Published:** 2026-10-26

**Authors:** Hui Qin, Tao Zeng, Lu Liu

**Affiliations:** Department of medicine, Jingchu University of Technology, Jingmen, China

**Keywords:** artificial intelligence readiness, influencing factors, large language model, nursing education, nursing students

## Abstract

**Objective:**

To investigate the current status of artificial intelligence (AI) readiness among nursing students and analyze its influencing factors, providing evidence for the cultivation of AI literacy in nursing education.

**Methods:**

A total of 684 nursing students from two universities in Hubei Province were recruited as study participants. Data were collected using a general demographic questionnaire and the Chinese version of the Medical Artificial Intelligence Readiness Scale for Medical Students (C-MAIRS-MS). Descriptive statistics, univariate analysis, and multiple linear regression analysis were employed for data processing.

**Results:**

The overall mean score of AI readiness among nursing students was 3.47 ± 0.31; the cognitive dimension scored the highest (3.57 ± 0.44), while the competency dimension scored the lowest (3.32 ± 0.48). Multiple linear regression analysis revealed that education level, Self-rated academic performance, Student cadre, Innovation and entrepreneurship project, Large language model use frequency, AI course learning, Programming foundation were significant predictors of AI readiness, collectively explaining 33.3% of the total variance (R^2^ = 0.333, adjusted R^2^ = 0.311, F = 15.030, *P* < 0.001)

**Conclusion:**

Nursing students’ AI readiness remains suboptimal with room for improvement. Targeted interventions addressing key predictors are essential to strengthen AI competency among nursing students.

## 1 Introduction

The rapid advancement of artificial intelligence (AI) technology is profoundly reshaping global healthcare delivery systems, ranging from intelligent diagnostic decision-support systems and nursing robots to electronic health record analytics and remote health monitoring, AI technology has permeated the entire spectrum of disease prevention, clinical diagnosis, rehabilitation nursing, and health management (1). In 2024, the World Health Organization (WHO) issued guidance on the ethics and governance of large multi-modal models for health. This guidance provides an international framework for the standardized use of AI in nursing education and clinical practice (2).

As the core force of the future nursing workforce, nursing students’ AI readiness directly determines the quality of intelligent nursing services and patient safety. Medical AI readiness is defined as healthcare providers’ readiness to effectively employ AI applications. It reflects their ability to integrate AI with professional expertise to deliver clinical services across prevention, diagnosis, treatment, and rehabilitation (3). In 2021, Karaca developed the Medical Artificial Intelligence Readiness Scale for Medical Students (MAIRS-MS) based on this concept, encompassing four dimensions: cognition, competency, vision, and ethics, providing a reliable instrument for systematically assessing medical students’ AI readiness (3). The scale has subsequently been cross-culturally validated in China (4), Iran (5), Malaysia (6), Saudi Arabia (7), Turkey (8), and Jordan (9), demonstrating excellent psychometric properties and cross-cultural applicability.

In recent years, scholars worldwide have conducted exploratory research on nursing students’ AI readiness. A survey of 227 nursing students found that students generally held a moderately positive attitude, although female students exhibited more negative attitudes toward AI than males (10). In another survey of 304 nursing students in Saudi Arabia, the ethics dimension scored the highest while the vision dimension scored the lowest, and educational level together with perceived AI advantages were found to be significant predictors of readiness (11). A quasi-experimental study in Turkey confirmed that a 14-week AI course intervention significantly improved nursing students’ total MAIRS-MS scores and their scores in the cognition, competency, and vision dimensions (12). Separately, a study of 386 Turkish nursing students revealed that AI readiness was significantly positively correlated with individual innovativeness, and that gender, family structure, income status, and AI training experience were all significant influencing factors (13).

In China, nursing education is undergoing a profound transformation from traditional skill-oriented to human-machine collaborative competency-oriented approaches. In 2024, the Ministry of Education reclassified nursing as a state-controlled specialty, signifying the nation's high regard for the quality of nursing talent cultivation (14). The Education Power Construction Plan Outline (2024–2035) explicitly calls for the deep integration of education with cutting-edge technologies such as AI and virtual reality to construct an intelligent educational ecosystem. However, in the nursing talent cultivation system, the curriculum for nursing informatics remains relatively underdeveloped, with insufficient integration of artificial intelligence technology (15).

This study aims to investigate the current status of AI readiness among nursing students and to analyze differences in AI readiness across students with different characteristics, thereby providing a reference for promoting AI education reform in nursing and formulating targeted strategies to enhance AI readiness.

## 2 Methods

### 2.1 Setting and participants

A convenience sampling method was employed to recruit nursing students from two universities in Hubei Province between April and May 2026.

Inclusion criteria were: (1) full-time nursing students currently enrolled in a degree program; and (2) voluntary participation with informed consent.

Exclusion criteria were: (1) students on leave of absence for more than one semester; and (2) students unable to complete the questionnaire due to personal reasons.

An *a priori* power analysis was conducted using GPower 3.1.9.2. Based on *F* tests for linear multiple regression (fixed model, R^2^ deviation from zero), with a medium effect size (f^2^ = 0.15), α = 0.05, power (1−β) = 0.95, and 44 predictors(22 demographic variables and 22 scale items), the minimum required total sample size was calculated to be 305. The actual sample size of 684 exceeded this requirement, achieving 95% statistical power and effectively minimizing the risk of Type II error, thereby ensuring the robustness of the findings.

### 2.2 Research instruments

#### 2.2.1 General demographic data

A self-designed questionnaire was developed by the researchers, including demographic characteristics (gender, age, educational level, grade, birthplace, family economic status, monthly living expenses), academic background (admission method, self-rated academic performance, student leadership experience, research and innovation projects, plans for postgraduate entrance examination or “top-up” degree program, internship status), and technical exposure experience (frequency of electronic device use, frequency of large language model use, AI course learning, nursing informatics-related courses, AI lectures, computer certificates or programming foundation, family IT background), comprising a total of 22 items.

#### 2.2.2 Chinese version of the medical artificial intelligence readiness scale for medical students (C-MAIRS-MS)

The C-MAIRS-MS, translated and validated by Tang et al. (16), was originally developed by Karaca et al. (3).The scale comprises four dimensions: cognition (8 items), competency (8 items), vision (3 items), and ethics (3 items), totaling 22 items. A 5-point Likert scale was used, ranging from 1 (strongly disagree) to 5 (strongly agree), with a total score range of 22–110. Higher scores indicate greater AI readiness.

In this study, the Cronbach’s *α* coefficient of the scale was 0.967, and the Cronbach’s *α* coefficients for each dimension were 0.920 (cognition), 0.929 (competency), 0.874 (vision), and 0.878 (ethics), indicating good reliability.

### 2.3 Data collection and quality control

This study employed both online and offline data collection. Online questionnaires were distributed via the Wenjuanxing platform with IP/device restrictions to prevent duplicate responses. Offline paper questionnaires were distributed by nursing teachers, who verified that participants had not previously completed the online version. The proportion of online versus offline responses was comparable between universities (∼70% online, 30% offline). While survey mode could theoretically influence responses, identical instructions and questionnaire content were used across both modes. Prior to the survey, trained investigators explained the research purpose, anonymity, and confidentiality. Questionnaires with completion times <180 s, obvious patterns, or inconsistencies were excluded.

### 2.4 Statistical analysis

Statistical analyses were conducted using SPSS 26.0. Continuous variables are presented as mean ± standard deviation, and categorical variables as frequencies and percentages. Independent samples t-tests and one-way ANOVA were used for between-group comparisons. Multiple linear regression was performed with the overall mean AI readiness score as the dependent variable and variables significant in univariate analysis as independent variables. Statistical significance was set at *α* = 0.05.

## 3 Results

### 3.1 General information of the study participants

A total of 723 questionnaires were distributed, and 684 valid questionnaires were returned, yielding an effective response rate of 94.6%. Thirty-nine questionnaires were excluded based on predetermined criteria: 17 for completion time <180 s, 12 for obvious response patterns (defined as identical responses across ≥80% of items or systematic alternating patterns), 7 for internal inconsistencies, and 3 for incompleteness (missing >10% of items). All exclusion criteria were established prior to data analysis and applied consistently by two independent researchers.

Among the 684 nursing students, 574 were female and 110 were male; their ages ranged from 17 to 25 (19.44 ± 1.15) years; 25.6% (175/684) had participated in AI course learning, and 47.8% (327/684) used large language models frequently or regularly to assist their learning, Table 1.

**Table 1:** Univariate analysis of demographic characteristics on artificial intelligence readiness (*n* = 684).

Variable	Category	*n*	Mean ± SD	t/F	*P*
Education level	Associate degree	340	3.35 ± 0.29	−10.961	＜0.001
	Bachelor degree	344	3.58 ± 0.28		
Grade	First year	206	3.39 ± 0.30	18.291	＜0.001
	Second year	190	3.43 ± 0.29		
	Third year	202	3.49 ± 0.32		
	Fourth year	86	3.66 ± 0.24		
Age(year)	≤18	177	3.38 ± 0.30	11.882	＜0.001
	19–21	487	3.49 ± 0.31		
	≥22	20	3.63 ± 0.21		
Gender	Male	110	3.56 ± 0.31	3.447	＜0.001
	Female	574	3.45 ± 0.30		
Place of origin	Urban	363	3.47 ± 0.30	0.782	0.217
	Rural	321	3.46 ± 0.32		
Family economic status	Difficult	73	3.41 ± 0.31	2.806	0.039
	Average	255	3.46 ± 0.31		
	Good	272	3.46 ± 0.30		
	Excellent	84	3.56 ± 0.31		
Monthly living expenses（RMB）	≤1,000	80	3.42 ± 0.29	1.965	0.118
	1,001–1,500	260	3.48 ± 0.31		
	1,500–2,000	235	3.52 ± 0.33		
	≥2,000	109	3.45 ± 0.30		
Admission method	National college entrance exam	466	3.52 ± 0.31	14.532	＜0.001
	Vocational college entrance exam	99	3.36 ± 0.27		
	Independent recruitment	102	3.35 ± 0.28		
	Other	17	3.34 ± 0.31		
Self-rated academic performance	Average	148	3.40 ± 0.30	6.921	0.001
	Good	386	3.46 ± 0.31		
	Excellent	150	3.54 ± 0.29		
Student cadre	Yes	106	3.55 ± 0.29	3.037	0.002
	No	578	3.45 ± 0.31		
Innovation and entrepreneurship project	Yes	93	3.61 ± 0.29	4.932	＜0.001
	No	591	3.44 ± 0.30		
Postgraduate entrance exam/Upgrading to undergraduate	Yes	282	3.48 ± 0.32	1.897	0.151
	No	225	3.48 ± 0.30		
	Uncertain	177	3.43 ± 0.23		
Currently interning	Yes	294	3.52 ± 0.32	3.781	＜0.001
	No	390	3.43 ± 0.30		
Electronic device usage frequency（hour)	<1	27	3.48 ± 0.33	0.131	0.942
	1–3	215	3.46 ± 0.29		
	3–5	300	3.47 ± 0.33		
	>5	142	3.48 ± 0.29		
Large language model	Never used	88	3.28 ± 0.28	33.354	＜0.001
	Occasionally	269	3.41 ± 0.29		
	Frequently	227	3.53 ± 0.29		
	Very frequently	100	3.65 ± 0.29		
AI course learning	Yes	175	3.58 ± 0.30	5.677	＜0.001
	No	509	3.43 ± 0.30		
Nursing informatics course	Yes	255	3.48 ± 0.31	0.633	0.531
	No	224	3.46 ± 0.31		
	Unclear	205	3.45 ± 0.30		
AI lecture	Yes	157	3.46 ± 0.31	−0.107	0.915
	No	527	3.47 ± 0.31		
Computer certificate or programming foundation	Yes	107	3.60 ± 0.29	5.125	＜0.001
	No	577	3.44 ± 0.30		
Family IT background	Yes	92	3.46 ± 0.32	−0.111	0.912
	No	592	3.47 ± 0.31		

AI = artificial intelligence.

### 3.2 Current status of AI readiness among nursing students

The results showed that the overall mean score of AI readiness among nursing students from the two participating institutions in Hubei Province was 3.47 ± 0.31, indicating a moderately high level. Among the four dimensions, the cognitive dimension scored the highest (3.57 ± 0.44), followed by the ethics dimension (3.52 ± 0.58) and the vision dimension (3.45 ± 0.64), while the competency dimension scored the lowest (3.32 ± 0.48), as shown in Table 2.

**Table 2:** Scores of artificial intelligence readiness (*n* = 684).

Dimension	Number of items	Mean ± SD	Rank
Cognition	8	3.57 ± 0.44	1
Ability	8	3.32 ± 0.48	4
Vision	3	3.45 ± 0.64	3
Ethics	3	3.52 ± 0.58	2
Artificial intelligence readiness	22	3.47 ± 0.31	

### 3.3 Univariate analysis of AI readiness by demographic characteristics

Univariate analysis results showed that educational level, grade, age, gender, family economic status, admission method, self-rated academic performance, student leadership experience, participation in innovation and entrepreneurship projects, internship status, frequency of large language model use, AI course learning, and computer certificates or programming foundation all had significant effects on nursing students’ AI readiness (*P* < 0.05), as shown in Table 1.

### 3.4 Multiple linear regression analysis of AI readiness influencing factors

Multiple linear regression analysis was conducted with the overall mean score of AI readiness as the dependent variable and variables with statistically significant differences in univariate analysis and five potential important confounding variables as independent variables. Ordinal categorical variables were treated as continuous variables, while nominal categorical variables were dummy-coded. Detailed variable assignments are presented in Table 3**.** The Enter method was employed for regression model construction, with potentially important confounders retained irrespective of univariate statistical significance. Interaction terms were not examined.

**Table 3:** Variable coding and assignment.

Variable	Category
Education level	1 = Associate degree；2 = Bachelor degree
Grade	1 = First year；2 = Second year；3 = Third year；4 = Fourth year
Age(year)	1=≤18；2 = 19∼21；3=≥22
Gender	1 = Male；2 = Female
Family economic status	1 = Difficult；2 = Average；3 = Good；4 = Excellent
Admission method	Reference：National college entrance exam (0,0,0,0)；Vocational college entrance exam(1,0,0,0)；Independent recruitment(0,1,0,0)；Other(0,0,1,0)
Self-rated academic performance	1 = Average；2 = Good；3 = Excellent
Student cadre	1 = Yes；2 = No
Innovation and entrepreneurship project	1 = Yes；2 = No
Postgraduate entrance exam/Upgrading to undergraduate	Reference：Yes(000)；No(1,0,0)；Uncertain(0,1,0)
Currently interning	1 = Yes；2 = No
Electronic device usage frequency（hour)	1=<1 ；2 = 1–3 ；3 = 3–5 ；4=>5
Large language model	1 = Never used；2 = Occasionally；3 = Frequently；4 = Very frequently
AI course learning	1 = Yes；2 = No
Nursing informatics course	Reference：Yes(0,0,0)；No(1,0,0)；Uncertain(0,1,0)
AI lecture	1 = Yes；2 = No
Computer certificate or programming foundation	1 = Yes；2 = No
Family IT background	1 = Yes；2 = No

Collinearity diagnostics showed that all variance inflation factors (VIF) were less than 4 (range: 1.021–3.685), and tolerance values were greater than 0.25 (range: 0.271–0.979), indicating no serious multicollinearity in the model.

Regression results revealed that education level, Self-rated academic performance, Student cadre, Innovation and entrepreneurship project, Large language model use frequency, AI course learning, Programming foundation entered the regression equation, collectively explaining 33.3% of the total variance (R^2^ = 0.333, adjusted R^2^ = 0.311, *F* = 15.030, *P* < 0.001), as shown in Table 4**.**

**Table 4:** Results of multiple linear regression analysis.

	Unstandardized coefficients		Standardized coefficient			95%CI		Collinearity statistics	
Variable	B	SE	Beta	t	P	LOW	UP	Tolerance	VIF
(Constant)	3.429	0.176		19.461	<0.001	3.083	3.775		
Education level	0.18	0.029	0.292	6.148	<0.001	0.122	0.237	0.448	2.233
Grade	0.002	0.018	0.007	0.116	0.908	−0.034	0.038	0.271	3.685
Age (years)	0.043	0.031	0.068	1.393	0.164	−0.018	0.105	0.419	2.389
Gender	−0.05	0.029	−0.060	−1.752	0.080	−0.106	0.006	0.873	1.146
Family economic status	0.011	0.012	0.029	0.898	0.369	−0.013	0.034	0.947	1.056
Self-rated academic performance	0.058	0.015	0.125	3.856	<0.001	0.029	0.088	0.958	1.044
Student cadre	−0.060	0.028	−0.07	−2.147	0.032	−0.114	−0.005	0.947	1.056
Innovation and entrepreneurship project	−0.090	0.03	−0.101	−2.967	0.003	−0.15	−0.031	0.879	1.138
Vocational college entrance exam	0.028	0.035	0.032	0.791	0.429	−0.041	0.097	0.629	1.59
Independent recruitment	0.026	0.035	0.030	0.752	0.452	−0.042	0.094	0.626	1.597
Other admission methods	0.073	0.067	0.037	1.096	0.274	−0.058	0.205	0.885	1.13
Currently interning	−0.006	0.027	−0.01	−0.223	0.824	−0.058	0.047	0.547	1.828
Electronic device usage frequency	0.005	0.012	0.012	0.378	0.706	−0.020	0.029	0.974	1.027
Large language model use frequency	0.096	0.011	0.279	8.427	<0.001	0.074	0.119	0.918	1.089
AI course learning	−0.114	0.023	−0.161	−4.879	<0.001	−0.159	−0.068	0.928	1.077
nursing informatics course(No)	0.046	0.024	0.070	1.910	0.057	−0.001	0.094	0.744	1.344
nursing informatics course(Unclear)	0.007	0.025	0.011	0.295	0.768	−0.041	0.055	0.757	1.32
AI lecture/training	0.045	0.024	0.061	1.858	0.064	−0.003	0.092	0.945	1.058
Programming foundation	−0.110	0.028	−0.13	−3.989	<0.001	−0.164	−0.056	0.953	1.049
Family IT background	−0.025	0.029	−0.028	−0.859	0.391	−0.082	0.032	0.979	1.021
postgraduate entrance exam/upgrading (No)	0.042	0.023	0.063	1.774	0.077	−0.004	0.088	0.789	1.267
postgraduate entrance exam/upgrading(Uncertain)	−0.017	0.025	−0.024	−0.685	0.493	−0.066	0.032	0.809	1.236

Dependent variable: Artificial intelligence readiness. (R^2^ = 0.333, adjusted R^2^ = 0.311, *F* = 15.030, *P* < 0.001.

The regression assumptions were assessed as follows. The scatter plot (Figure 1) of standardized predicted values against standardized residuals showed random dispersion without discernible patterns, supporting the linearity and homoscedasticity assumptions. Normality was confirmed by the normal P-P plot **(**Figure 2), in which the points closely adhered to the diagonal, and by the histogram, which corresponded well with the normal curve (Figure 3). Independence of errors was supported by a Durbin-Watson statistic of 1.918, which is close to 2 and indicates no autocorrelation. Multicollinearity was not a concern, as all variance inflation factors (VIF) were below 4 (range: 1.021–3.685) and all tolerance values exceeded 0.25. Standardized residuals ranged from −2.996 to 2.965, with only a few cases marginally exceeding ±3; given the large sample size (*N* = 684), these cases did not materially affect the model.

**Figure 1:**
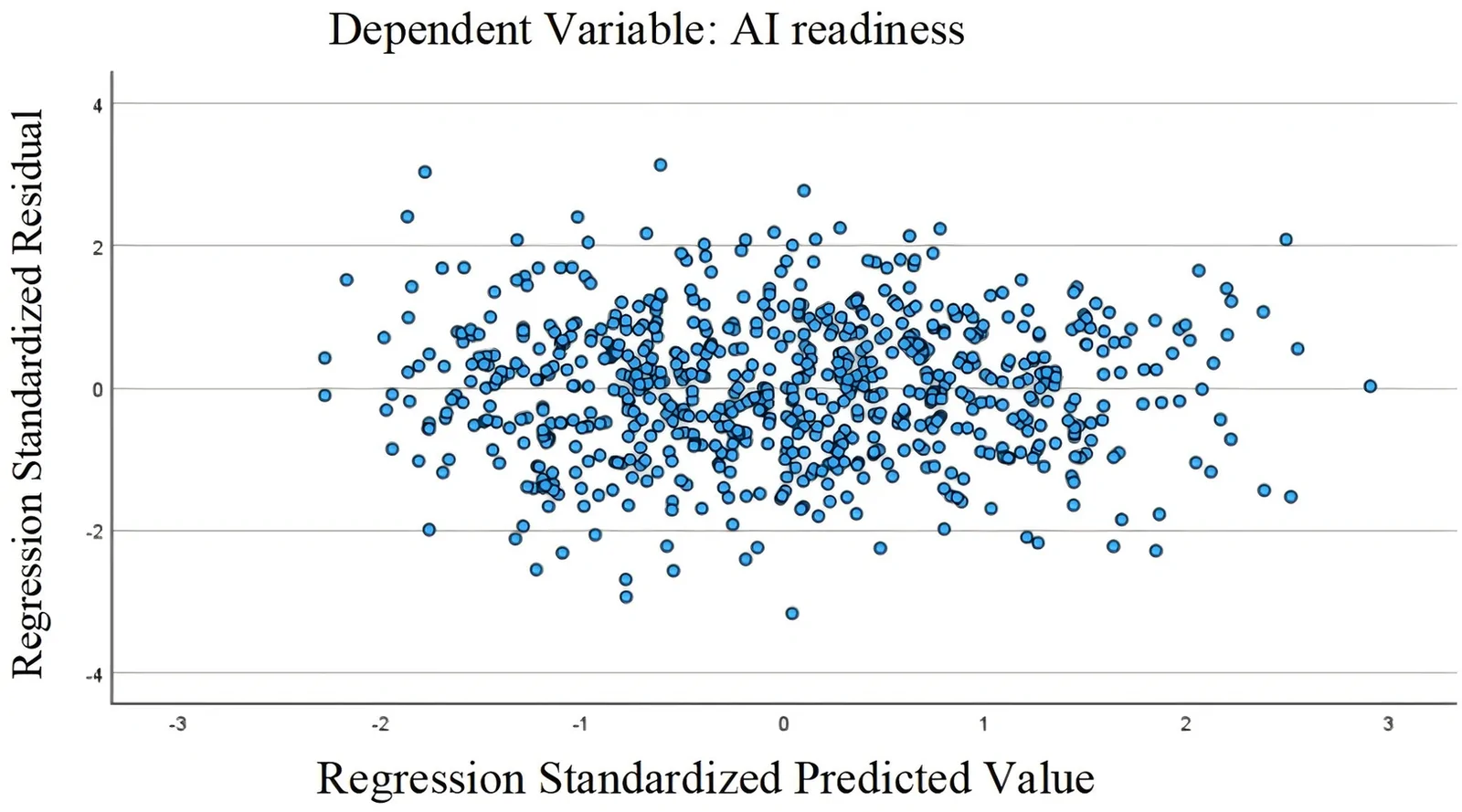
Scatterplot.

**Figure 2:**
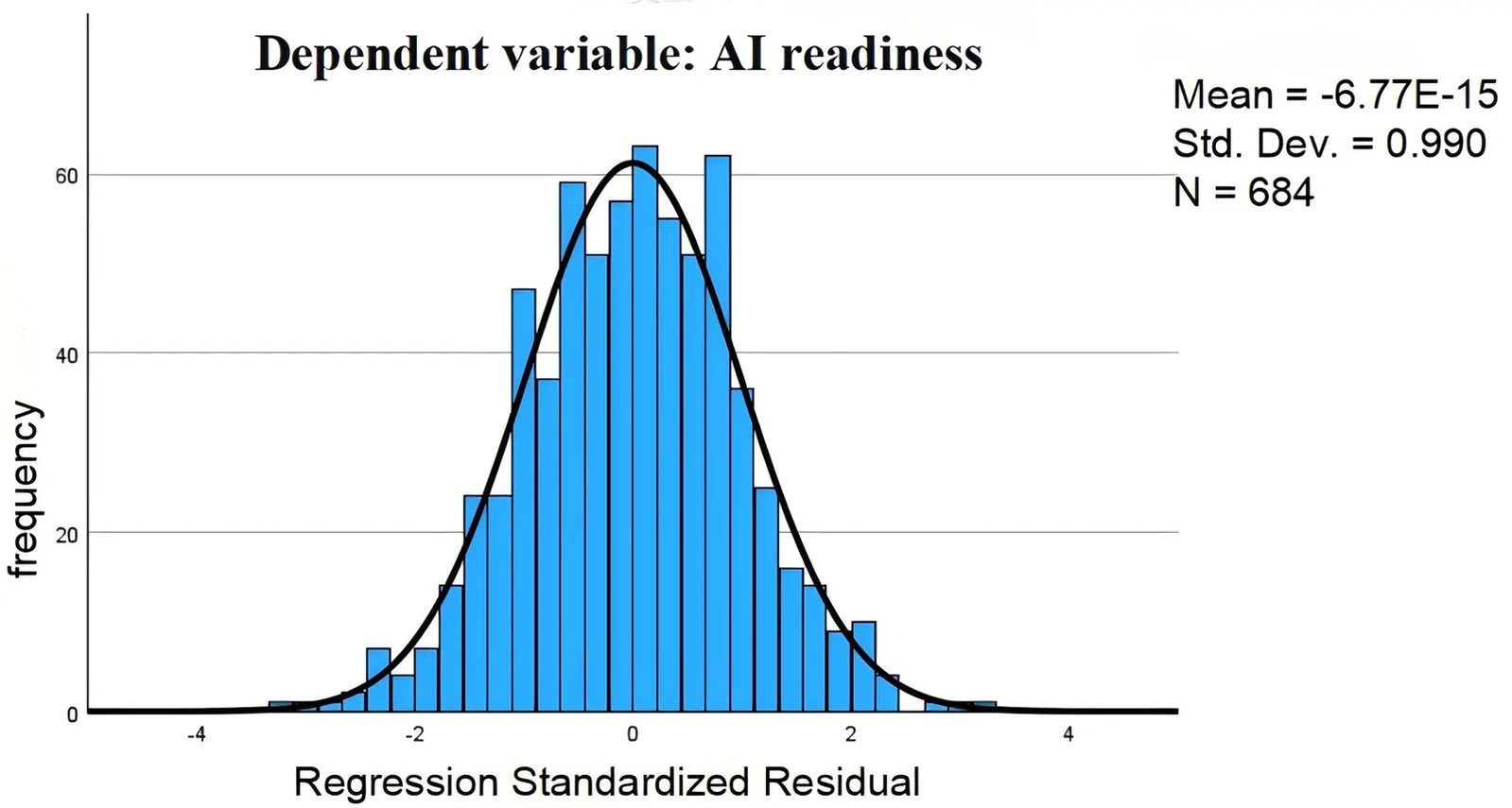
Histogram.

**Figure 3:**
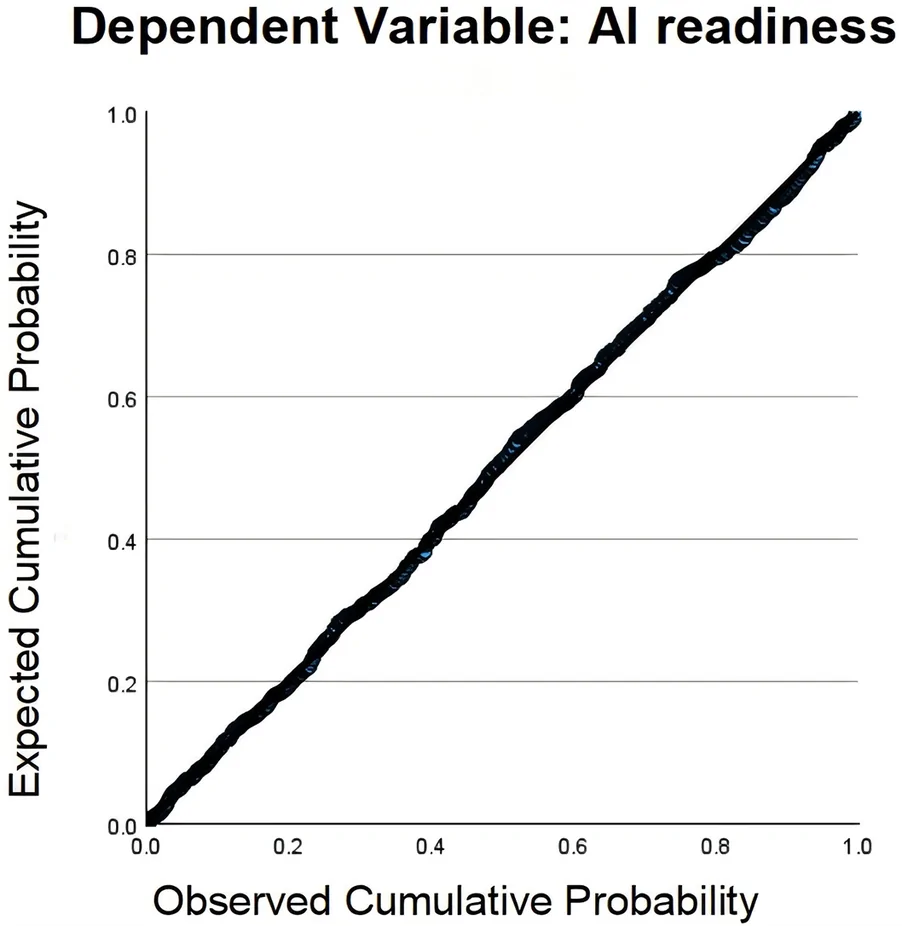
Normal P-P plot of regression standardized residual.

Regression diagnostics indicated no obvious violations of key model assumptions.

## 4 Discussion

### 4.1 Current status of AI readiness among nursing students

The results of this study showed that the overall mean score of AI readiness among nursing students from the two participating institutions in Hubei Province was 3.47 ± 0.31. Among the four dimensions, the cognitive dimension scored the highest (3.57 ± 0.44), followed by the ethics dimension (3.52 ± 0.58) and the vision dimension (3.45 ± 0.64), while the competency dimension scored the lowest (3.32 ± 0.48). These findings are generally consistent with the survey results of Previous studies among Chinese medical students (16, 17).

The core reason lies in the structural deficiency of emphasizing theory over practice in the education system, with a clear readiness-impact gap (18), the penetration rate of AI courses is insufficient (only 25.6% of students participated), and students’ use of large language models mostly remains at a superficial query-based level (occasional and never users accounted for 52.2%), lacking systematic learning by doing training. Additionally, the traditional skill-oriented curriculum inertia in nursing, insufficient demonstration of AI technology during clinical internships, and lower technical self-efficacy among vocational-level students and female students collectively create barriers to the transformation from knowing AI to using AI.

### 4.2 Differences in AI readiness among nursing students with different characteristics

Multiple linear regression analysis results showed that educational level was a positive predictor of AI readiness (*B* = 0.180, SE = 0.029, β = 0.292, 95% CI (0.122, 0.237), *t* = 6.148, *P* < 0.001), with undergraduate students (3.58 ± 0.28) scoring significantly higher than vocational students (3.35 ± 0.29). Undergraduate education typically includes more comprehensive information technology courses, research training, and thesis requirements, which cultivate students’ information retrieval, data processing, and critical thinking abilities, prerequisites for effective use of AI tools. Gandhi confirmed the positive correlation between educational level and AI readiness (19). However, it is noteworthy that after controlling for other variables, grade and age did not enter the final regression model, suggesting that educational level itself is a stronger predictor of AI readiness than grade or age. This implies that the lower AI readiness of vocational-level students is not due to lower grade but rather the lack of systematic AI literacy training in their overall cultivation programs. Therefore, universities should construct a stratified and progressive AI literacy cultivation system, providing differentiated support pathways for vocational-level students to avoid the widening of the technology gap exacerbating educational inequality (20).

Self-rated excellent students demonstrated significantly higher readiness than self-rated average students (*B* = 0.058, SE = 0.015, β = 0.125, 95% CI (0.029, 0.088), *t* = 3.856, *P* < 0.001). Possible explanations may be that students with higher academic self-efficacy typically possess stronger learning motivation, better metacognitive strategies, and higher achievement goal orientation, which transfer to AI learning as more active technology exploration behaviors and more efficient learning transfer abilities. Eltantawy (21) confirmed in a study of 550 Egyptian nursing students that academic ability was significantly positively correlated with AI attitude, with academic qualification having a partial mediating effect. High-achieving students are often more adept at utilizing AI tools for literature retrieval, knowledge integration, and self-directed learning, forming a virtuous cycle of academic success to technical confidence to higher readiness. It is recommended to implement differentiated teaching strategies, providing differentiated AI learning scaffolds for students at different academic levels (5).

Frequency of large language model use exhibited the largest standardized coefficient (β = 0.279) among all predictors of AI readiness. (*B* = 0.096, SE = 0.011, β = 0.279, 95% CI (0.074, 0.119), *t* = 8.427, *P* < 0.001). However, given the cross-sectional design, this association should not be interpreted as causal; reverse causation is plausible, as students who are already more AI-ready may simply use LLMs more frequently. Students who never used large language models accounted for 12.9% (88/684), and occasional users accounted for 39.3% (269/684), indicating that a considerable proportion of students remain in a digital bystander state. Sarkar et al. (22) confirmed in a study of 362 medical students that familiarity with AI terminology and AI training experience were significant positive predictors of AI attitude, corroborating our findings. Nursing teachers should design immersive, task-driven learning experiences to facilitate the transition from occasional use to deep integration, incorporating large language models into real task scenarios such as nursing assessment, health education, and literature review (23).

Students who received systematic AI course learning demonstrated higher readiness,which is consistent with the findings of Taskiran (12). In this study, only 25.6% (175/684) of students participated in AI course learning, a proportion that, while higher than some international reports, still reflects the reality of insufficient curriculum supply. Al Shahrani et al. (7) reported that only 14.5% of Saudi medical students received formal AI education; A study showed that 95.3% of medical and health sciences students had not obtained any AI education experience from their schools. The results of this study strongly call for nursing institutions to elevate AI courses from elective to required status and adopt a spiral curriculum design, integrating AI fundamentals, applications, and ethics content throughout nursing core courses.

Students with computer certificates or programming foundations demonstrated significantly higher readiness than those without (*B* = −0.110, SE = 0.028, β = −0.130, 95% CI (−0.164, −0.056), *t* = −3.989, *P* < 0.001). Programming training not only cultivates students’ computational thinking but more importantly establishes a human-machine collaborative work mental model, understanding algorithm logic, data input-output relationships, and technological limitations. However, in this study, only 15.6% (107/684) of students possessed programming foundations, which may be reflecting the general lack of computational thinking training among nursing students. Nursing education traditionally emphasizes humanistic care and clinical skills, with insufficient attention to technical literacy. It is recommended that nursing institutions add modules such as Fundamentals of Nursing Informatics and Introduction to Health Data Analysis to their curriculum, utilizing low-code or no-code platforms to lower learning barriers.

Studen cadre, (*B* = −0.060, SE = 0.028, β = −0.070, 95% CI (−0.114, −0.005), *t* = −2.147, *P* = 0.032) and participation in innovation and entrepreneurship projects (*B* = −0.090, SE = 0.030, β = −0.101, 95% CI (−0.150, −0.031), *t* = −2.967, *P* = 0.003) both significantly affected AI readiness. Possible explanations may be that Student cadre typically undertake more organizational coordination, information integration, and decision-making tasks, experiences that cultivate their information processing and problem-solving abilities, making them more adept at utilizing AI tools to assist management decisions and team collaboration. Innovation and entrepreneurship project experience promotes the application of AI tools to practical problem-solving, such as using AI for literature review, data analysis, or health intervention program design, thereby deepening technical understanding and establishing interdisciplinary thinking.

Kaya et al. (13) revealed that AI readiness was significantly positively correlated with individual innovativeness, with innovation and entrepreneurship training indirectly promoting AI readiness by enhancing students’ innovative self-efficacy. This suggests that nursing education should construct a trinity AI literacy cultivation system integrating curriculum, research, and competition: embedding AI application cases in course teaching, introducing AI-assisted data analysis in research training, and setting up intelligent nursing special tracks in innovation and entrepreneurship competitions, forming a multi-dimensional, immersive AI learning ecosystem (11).

## 5 Limitations

This study adopted a cross-sectional design, which precludes the establishment of causal relationships between variables. Although the multiple regression model explained 31.1% of the variance, a large number of unidentified factors (such as school AI infrastructure, faculty digital literacy, and family technology environment) may also influence students’ AI readiness. The sample of this study was drawn from two universities in HuBei province, and generalizability is limited. Multi-center, large-sample studies are recommended to validate the conclusions of this study.

Furthermore, The C-MAIRS-MS was originally designed for medical students, and while the Chinese version has demonstrated excellent internal consistency in our nursing sample, its construct validity has not been specifically established among nursing students.

This study relied exclusively on self-reported measures, which may introduce social desirability bias, recall bias, and common-method variance. The observed associations between self-rated academic performance, AI exposure, and AI readiness may be partly inflated due to shared method variance. Future studies should incorporate objective measures (e.g., actual AI usage logs, academic records) and employ methodological remedies such as temporal separation of predictor and outcome measurements to mitigate these biases.

## 6 Conclusion

This study found that nursing students’ AI readiness was (3.47 ± 0.31). China's Education Power Construction Plan Outline (2024–2035) explicitly calls for the deep integration of education with cutting-edge technologies such as AI and virtual reality to construct an intelligent educational ecosystem, which echoes the cognitive, competency, vision, and ethical dimensions of AI readiness and reveals the strategic significance of enhancing nursing students’ AI readiness in cultivating their AI literacy. Nursing educators need to further optimize the design of AI general education courses, integrate AI technology throughout the entire teaching and learning process, and focus on improving nursing students’ (especially vocational-level students, female students, lower-grade students, those with lower self-rated academic performance, and those with lower frequency of LLM-assisted learning) cognitive depth, application competency, and ethical judgment of AI technology, systematically enhancing their AI readiness.

## Data Availability

The original contributions presented in the study are included in the article/Supplementary Material, further inquiries can be directed to the corresponding authors.
